# GASOLINE: a Cytoscape app for multiple local alignment of PPI networks

**DOI:** 10.12688/f1000research.4537.2

**Published:** 2014-09-23

**Authors:** Giovanni Micale, Andrea Continella, Alfredo Ferro, Rosalba Giugno, Alfredo Pulvirenti

**Affiliations:** 1 Department of Computer Science, University of Pisa, Pisa, 56127, Italy; 2 Department of Electronics, Information and Bioengineering, Polytechnic University of Milan, Milan, 20133, Italy; 3 Department of Clinical and Molecular Biomedicine, University of Catania, Catania, 95125, Italy

## Abstract

Comparing protein interaction networks can reveal interesting patterns of interactions for a specific function or process in distantly related species. In this paper we present GASOLINE, a Cytoscape app for multiple local alignments of PPI (protein-protein interaction) networks. The app is based on the homonymous greedy and stochastic algorithm. GASOLINE starts with the identification of sets of similar nodes, called seeds of the alignment. Alignments are then extended in a greedy manner and finally refined. Both the identification of seeds and the extension of alignments are performed through an iterative Gibbs sampling strategy. GASOLINE is a Cytoscape app for computing and visualizing local alignments, without requiring any post-processing operations. GO terms can be easily attached to the aligned proteins for further functional analysis of alignments. GASOLINE can perform the alignment task in few minutes, even for a large number of input networks.

## Introduction

In the last few years there has been a rapid growth of biological network data, including protein-protein interaction (PPI) networks, metabolic networks and regulatory networks ^1– 6^. Among these, PPI networks are important in several biological phenomena such as signaling, transcriptional regulation and formation of multi-enzyme complexes ^7^.

Comparing PPI networks of evolutionary distant species can help to understand the regulation of a specific function or process, which the sequence comparison alone cannot explain ^8^. Network alignment detects functionally orthologous proteins across networks of different species. Alignment methods can be local or global. In local alignment, networks are compared in order to find conserved protein complexes or pathways ^8– 10^. Global alignment methods ^10– 13^ try to find a complete one-to-one mapping between functional orthologous to infer evolutionary relationships among different species.

Most network alignment algorithms have been provided with their implementations, however none of them is fully integrated within Cytoscape. Cytoscape ^14^ is an open source platform for visualizing and analyzing molecular interaction networks and integrating data with gene expression profiles and other kind of knowledge. Cytoscape functionality can be easily extended through Java apps.

Here, we describe a Cytoscape app based on GASOLINE (Greedy And Stochastic algorithm for Optimal Local alignment of Interaction NEtworks), a greedy and stochastic algorithm for multiple local alignment of PPI networks, introduced in ^15^. We chose to build a Cytoscape app for GASOLINE because Cytoscape is a very popular tool in bioinformatics and is written in Java, therefore multi-platform.

The algorithm starts by identifying a set of similar nodes, one from each network, by using a Gibbs Sampling strategy ( *bootstrap phase*). Then, a similar technique is applied in the *iterative phase*, where the alignment is extended until the local density of the aligned subgraphs increases. A single extension step consists of adding one node from each network to the current subgraph alignment. Finally, in the *refinement phase*, the alignment is refined by iteratively removing aligned nodes and adding new mapped ones.

GASOLINE is very accurate and scalable with respect to the number of aligning networks. However, it produces a one-to-one mapping between aligned nodes and its running time is moderately affected by the size and the average density of input networks.

The app enables the 2D visualization of local alignments and aligned proteins can be associated with GO annotations for further functional analysis. To the authors knowledge, it is the first Cytoscape tool for the computation and visualization of local alignments in a user-friendly way, without requiring any post-processing operations.

## Implementation

GASOLINE app encloses the original Java implementation of the namesake algorithm ^15^, with the addition of a visualization module, which exploits the Cytoscape interface.

The app has been written in Java version 7 and designed following a classic Model-View-Controller (MVC) model. The Model part is represented by the classes implementing the algorithm and the auxiliary data structures. The View part is composed by two Java Panels; one for setting all the input and output parameters, and one for listing local alignments and handling their visualization.

The Controller part ensures the communication between the Model and the View and is implemented by different Cytoscape Task classes, one for each process performed by GASOLINE (i.e. checking file format, computing alignments, importing networks, protein description and GO annotations, building the alignment graphs). Each Task class properly notifies the corresponding view class when a task has been completed.

Input networks are imported as text files and then internally represented in two different ways to optimize the performance of our algorithm. We used CyNetwork and CyNetworkView objects for network alignment visualization and custom classes for computing alignments. For all the imported networks, the corresponding Cytoscape view is initially disabled to reduce the memory consumption.

**Figure 1.**
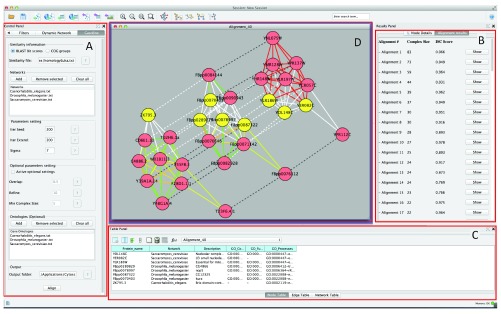
GASOLINE Cytoscape panels: **A**) GASOLINE parameters; **B**) Alignment results; **C**) Description of selected proteins and associated GO terms; **D**) Alignment visualization: intra-edges are represented with solid lines and coloured according to their weight (green for low values, yellow for medium values and red for high values); inter-edges are drawn with dashed lines.

The main component of GASOLINE is represented by a tabbed panel named “GASOLINE” located in the Control Panel of Cytoscape (see Figure 1, panel A). Through the interface the users can provide the following information:

- “Similarity information”, to upload orthology similarity scores between proteins of different species;
- “Networks”, for selecting two or more networks to align;
- “Parameters setting”, to modify the default GASOLINE parameters;
- “Optional parameters setting”, for setting other advanced input parameters;
- “Ontologies”, to upload GO terms linked to the proteins of the aligned networks;
- “Output”, to specify the folder where the final alignments will be saved.

The button labeled “?”, when present, explains the meaning of a specific function or parameter of GASOLINE, whenever the mouse arrow hovers.

In the following subsections, we will describe all the required steps to run GASOLINE on a set of PPI networks.

### Loading input data

Before running GASOLINE, the user needs to upload input data, including:

a) Two or more networks to be aligned;
b) A file of orthology BLAST bit scores between proteins of different species;
c) A set of GO terms linked to the proteins of each network.

The GO terms file is not mandatory and can be omitted.

Networks are given as a list of weighted edges and can be uploaded from the “Networks” panel. There is no theoretical limitation on the size and the number of uploadable networks.

Orthology data can be uploaded through the “Similarity information” panel. They can be supplied in two different formats: “BLAST Bit scores” or “COG groups”.

The “BLAST Bit scores” format is a text file where each row has a couple of proteins of different species followed by their corresponding BLAST bit score.

Files in the “COG groups” associate a list of orthology groups to the proteins of aligning networks. Groups include KOG (euKaryotic Orthologous Groups), NOG (Non-supervised Orthologous Group) and COG (Cluster of Orthologous Groups) terms. When aligning many networks, the “COG groups” format can be more suitable, since the number of possible pairwise bit scores grows exponentially with the number of aligning networks.

GO categories can be optionally uploaded from “Ontologies” panel. They are provided as text files, where a list of GO cellular components, processes and functions is associated to each protein. Note that GO terms are not used for computing the alignments and can only facilitate the understanding of the alignments returned by GASOLINE.

Whenever the GO categories are provided as input, the list of GO terms for a specific protein is added as node attribute in Cytoscape, these are accessible from the “Node Browser” tabbed panel once GASOLINE ends the computation and the local alignments are ready to be visualized.

### Setting the parameters

The main input GASOLINE parameters are specified in the “Parameters setting” panel. These include:

- “Iter Seed”: the number of iterations of Gibbs sampling in the bootstrap phase;
- “Iter Extend”: the number of iterations of Gibbs sampling in each extension step of the iterative phase;
- “Sigma”: minimum network degree of nodes that can be selected as seeds in the bootstrap phase.

Values for “Iter Seed” and “Iter Extend” depend on the number of aligning networks: the more networks we have, the higher these values should be. However, based on the experiments performed on real PPI networks and reported in ^15^, we empirically established that 200 iterations of Gibbs sampling in both phases are enough to produce reliable results for up to 25 networks.

The choice of “Sigma” implies a trade-off between speed and accuracy of GASOLINE: the higher the *σ*, the faster is GASOLINE but the lower is accuracy. If networks are very sparse (like most of the existing PPI networks), low values of sigma (1 or 2) are recommended.

The “Optional parameters setting” panel contains three more input parameters:

- “Overlap”: a value between 0 and 1, denoting the maximum allowed fraction of common nodes between two alignments, in order to be considered distinct. If two alignments have many nodes in common, the one with the least number of nodes is discarded from the final set;
- “Refine”: the number of iterations of GASOLINE in the refinement phase;
- “Min Complex Size”: the minimum size of conserved complexes in the final set of local alignments.

These parameters can be modified by checking the box “Active optional settings”, otherwise the default values will be used.

Note that a high value of the “Refine” parameter can be used to increase the accuracy of the local alignments, but the algorithm will be more time consuming. In our tests ^15^, we experienced that a value of 10 guarantees the best trade-off between speed and accuracy of GASOLINE.

For the “Overlap” and “Min Complex Size” parameters, we suggest 0.5 and 5 as default values, respectively.

Finally, the user can specify an output folder for the final alignments, by clicking on the text field next to the “Output folder” label. Each local alignment will be stored in a separate text file into the specified folder, containing the list of aligned sub-graphs and the one-to-one mapping between aligned nodes.

### Running GASOLINE

Once all the required input files are provided and all the parameters are set up, GASOLINE can be executed by clicking on the “Align” button. Then, a progress bar will appear.

### Visualizing local alignments

When GASOLINE ends, a table containing all the computed local alignments is shown on the right side of the “Results panel” of Cytoscape (see Figure 1, panel B). The table reports, for each alignment, the size of the aligned complexes and the ISC (Index of Structural Conservation) score. ISC index measures the average degree of structural conservation of an alignment and can be used to evaluate its quality.

Each row of the table contains a “Show” button, for the visualization of the corresponding alignment graph on the left side of the “Results Panel” of Cytoscape (see Figure 1, panel D).

In the alignment graph, each node is labeled with the ID of the corresponding protein. If the GO annotations have been provided, the user can select one or more nodes and view the description of the proteins and their corresponding GO terms (see Figure 1, panel C).

Two kinds of edges are shown (panel D):

- Intra-edge, linking proteins of the same network, which are represented with solid colored lines;
- Inter-edge, linking proteins of different networks in the local alignment, which are drawn with dashed lines.

Colors of intra-edges depend on the probability *p* of the corresponding protein-protein interaction: for low values of *p* colors range from green to yellow, for high values of *p* colors range from yellow to red. Weights are automatically associated to edges as attributes, therefore the user can select an edge and retrieve its weight from the “Edge Attribute Browser” (see Figure 1, panel C).

Layout “Kamada-Kawai” has been used for the visualization of the alignment graph.

## Results

Finally, we show an example of the workflow, using three well known biological networks ( *C. elegans*, *D. melanogaster*, *S. cerevisiae*) taken from the STRING database ^3^, considering only experimentally validated interactions. We also annotated proteins by using a set of GO annotations and protein descriptions taken from BioDBnet ^16^. This test was performed in a Intel Core i7-2670 2.2Ghz CPU with a RAM of 8 GB.

Following the steps described in the Implementation section, we loaded the three networks and ran GASOLINE, using the default parameters ( *IterSeed* = 200, *IterExtend* = 200, *Sigma* = 7, *Overlap* = 0.5, *Refine* = 10, *MinComplexSize* = 5).

**Figure 2.**
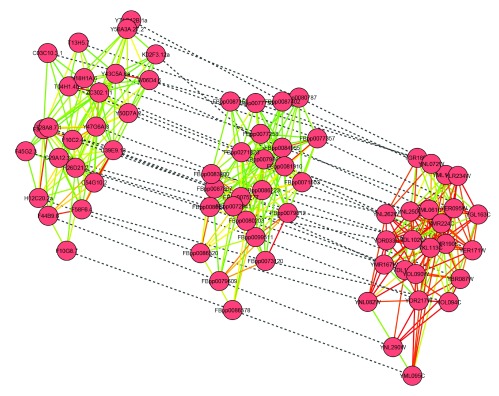
Alignment of DNA repair complex in *C. elegans*, *D. melanogaster* and *S. cerevisiae*.

GASOLINE took 110 seconds to complete the task and returned many known conserved complexes with a high degree of topological conservation (ISC between 80 and 90%). These include the large and small subunits of ribosomes (64 proteins), a serine/threonine kinase complex (34), the spliceosome (28), a DNA repair complex (24 proteins, Figure 2), the V-ATPase complex (17) and the ARP2/3 complex (16).

## Conclusions

In this paper, we presented GASOLINE, an app for Cytoscape 3 for computation and visualization of multiple local alignments of protein-protein interaction networks. To the best of our knowledge, it is the first Cytoscape plugin for computation and visualization of multiple local alignment of biological networks.

GASOLINE offers a user-friendly interface and an easy 2D visualization of local alignments. Moreover, alignments can be further investigated, by attaching GO terms to the proteins of aligning networks.

We believe that GASOLINE can be a valuable tool to better understand the functioning of some biological processes. It can help to transfer functional annotations from one species to another, to predict new complexes or to help in establishing the function of unknown human proteins within the cells.

## Software availability

The GASOLINE app, as well as datasets of real PPI networks and orthology files that can be directly used to run the algorithm, can be downloaded from the GASOLINE website: http://ferrolab.dmi.unict.it/gasoline/gasoline.html.

The GASOLINE plugin can also be downloaded from the Cytoscape App Store: http://apps.cytoscape.org/apps/gasoline.

On our website, there is also a complete documentation on the GASOLINE plugin, with more details about the format of input and output data, and a JAR file for running our algorithm in local with any platform.

Latest source code: https://github.com/GMicale/GASOLINE

Source code as at the time of publication: https://github.com/F1000Research/GASOLINE/releases/tag/V1.0

Archived source code as at the time of publication: http://www.dx.doi.org/10.5281/zenodo.104629
