# Supplementary material for: GASOLINE: a Cytoscape app for multiple local alignment of PPI networks
Source: F1000Res. 2014 Sep 23;3:140. Originally published 2014 Jul 1. [Version 2] doi: 10.12688/f1000research.4537.2 (PMC4197741; doi:10.12688/f1000research.4537.2)
Supplement: Peer review history

# Peer review: Referee response for version 2

**DOI:** 10.5256/f1000research.5731.r5293

**Published:** 2014-09-26

**Authors:** Antonio J Perez Pulido, Pablo Mier

**Affiliations:** 1 UPO Genetics Bioinformatics Group, Centro Andaluz de Biología del Desarrollo (CABD), Sevilla, Spain; 2 Universidad Pablo de Olavide, Sevilla, Spain

We are agreed with the last sent changes, and we think that the article can be indexed in its current state.

We have read this submission. We believe that we have an appropriate level of expertise to confirm that it is of an acceptable scientific standard.

# Peer review: Referee response for version 1

**DOI:** 10.5256/f1000research.4854.r5295

**Published:** 2014-07-15

**Authors:** Byung-Jun Yoon

**Affiliations:** 1 Electrical and Computer Engineering, Texas A&M University, College Station, TX, USA

In this paper, the authors introduce a Cytoscape plug-in that can be used to perform local alignments of protein-protein interaction (PPI) networks using GASOLINE. The local network algorithm algorithm called GASOLINE was developed by the same team and it was presented in a separate journal paper. As the authors claim, a Cytoscape app for the alignment and visualization of PPI networks may serve as a useful tool for researchers working with multiple PPI networks. However, there are also several issues that need to be properly addressed to make the paper more accessible and informative for potential readers.

**Abstract:**

- Readers may not be familiar with GASOLINE. Please provide a more detailed description of the algorithm.

**Introduction:**

-  "In the last few years there has been a rapid growth of biological network data, including protein-protein interaction (PPI) networks, metabolic networks and regulatory networks."
  Please add references for available PPI databases and resources.
- "Comparing PPI networks of evolutionary distant species can help to understand some mechanisms underlying a specific function or process, which the sequence comparison alone cannot explain."
  Please provide an example or cite a reference that shows when a simple sequence comparison does not suffice.
- "Local network alignment aims to compare networks of different species, in order to find conserved protein complexes or pathways."
  Define network alignment and briefly describe and compare different types of network alignments (e.g. local vs. global).
- "In literature, several network alignment algorithms have been described together with their implementations"
  At least a brief description of the popular existing methods would be helpful.
- "Here, we describe a Cytoscape app implementing the GASOLINE algorithm for multiple local alignment of PPI networks."
  Please provide at least a short introduction to GASOLINE. A brief overview of the algorithm and its pros/cons would be useful.
  Please explain what type of local alignment is obtained by GASOLINE. (e.g., one-to-one vs. many-to-many).

**Implementation**

- Define acronyms before use (e.g, COG, KOG, NOG, ISC)
- "The “COG groups” format can be more convenient when aligning many networks since the all the possible pairwise bit scores are many."
  Meaning unclear. Please revise.
- "GO categories can be optionally uploaded from “Ontologies” panel."
  What are the added benefits of uploading GO information? Are they used in any way during the alignment? Does it provide additional information regarding the predicted local alignments? If it does, what kind of extra info can be obtained by uploading GO files?
- Please describe the capabilities and limitations of the developed Cytoscape app on a typical personal computer. How many networks can be handled by the GASOLINE Cytoscape plug-in? How about the maximum size of the networks? (e.g., number of nodes, number of interactions). How does it scale with the number (size) of networks?
- Please describe the main difference between the original GASOLINE and the GASOLINE Cytoscape app discussed in this paper. Are there any technical differences? Are there any differences in terms of their capabilities/limitations? Any recommendation on when one should use the desktop version instead of the online version?

**Conclusions**

- Please discuss potential applications of the Cytoscape app.

I have read this submission. I believe that I have an appropriate level of expertise to confirm that it is of an acceptable scientific standard, however I have significant reservations, as outlined above.

# Response

**Published:** 2014-09-23

**Authors:** Giovanni Micale

We thank the reviewer for his helpful comments and suggestions.

- ABSTRACT

***Readers may not be familiar with GASOLINE. Please provide a more detailed description of the algorithm.***

We added a paragraph to the Abstract section, providing a more detailed description of GASOLINE.

- INTRODUCTION

***"In the last few years there has been a rapid growth of biological network data, including protein-protein interaction (PPI) networks, metabolic networks and regulatory networks."***

***Please add references for available PPI databases and resources.***

***"Comparing PPI networks of evolutionary distant species can help to understand some mechanisms underlying a specific function or process, which the sequence comparison alone cannot explain."***

***Please provide an example or cite a reference that shows when a simple sequence comparison does not suffice.***

We added all the references.

***"Local network alignment aims to compare networks of different species, in order to find conserved protein complexes or pathways."***

***Define network alignment and briefly describe and compare different types of network alignments (e.g. local vs. global).***

We included a small paragraph describing the network alignment problem and the differences between local and global alignment methods.

***"In literature, several network alignment algorithms have been described together with their implementations"***

***At least a brief description of the popular existing methods would be helpful.***

We do not give details of most important network alignment methods, but we added references to them.

***"Here, we describe a Cytoscape app implementing the GASOLINE algorithm for multiple local alignment of PPI networks."***

***Please provide at least a short introduction to GASOLINE. A brief overview of the algorithm and its pros/cons would be useful.***

***Please explain what type of local alignment is obtained by GASOLINE. (e.g., one-to-one vs. many-to-many).***

We provided more details concerning GASOLINE algorithm and all its phases. Moreover, we added a new paragraph, which highlights the virtues and the current limitations of GASOLINE. In this paragraph we also stress that GASOLINE produces a one-to-one mapping.

- IMPLEMENTATION

***Define acronyms before use (e.g, COG, KOG, NOG, ISC).***

We defined all the acronyms.

***"The “COG groups” format can be more convenient when aligning many networks since the all the possible pairwise bit scores are many."***

***Meaning unclear. Please revise.***

We stressed the benefits of using the "COG groups" format in the alignment of many networks.

***"GO categories can be optionally uploaded from “Ontologies” panel."***

***What are the added benefits of uploading GO information? Are they used in any way during the alignment? Does it provide additional information regarding the predicted local alignments? If it does, what kind of extra info can be obtained by uploading GO files?***

We clarified that GO information are never used by GASOLINE in the alignment task. They only give, when provided by the user, useful information to better understand the final set of local alignments returned by GASOLINE.

***Please describe the capabilities and limitations of the developed Cytoscape app on a typical personal computer. How many networks can be handled by the GASOLINE Cytoscape plug-in? How about the maximum size of the networks? (e.g., number of nodes, number of interactions). How does it scale with the number (size) of networks?***

We specified that theoretically there is no limit on the number and size of input networks. At the end of the “Introduction” section we specified that the running time of GASOLINE is moderately affected by the size and the average density of input networks, but it is scalable with the number of aligning networks.

***Please describe the main difference between the original GASOLINE and the GASOLINE Cytoscape app discussed in this paper. Are there any technical differences? Are there any differences in terms of their capabilities/limitations? Any recommendation on when one should use the desktop version instead of the online version?***

At the beginning of this section we underlined that there is no technical difference between the original GASOLINE and the Cytoscape App implementation. The Cytoscape App of GASOLINE wraps the original algorithm and simply includes a visualization module. So overall, the two versions are substantially equivalent.

- CONCLUSIONS

***Please discuss potential applications of the Cytoscape app.***

In the last paragraph of this section we sketch some interesting applications of our app, concerning annotation transfer and protein function prediction.

# Peer review: Referee response for version 1

**DOI:** 10.5256/f1000research.4854.r5342

**Published:** 2014-07-15

**Authors:** Rintaro Saito

**Affiliations:** 1 School of Medicine, University of California San Diego, La Jolla, CA, USA

I was unable to install the App GASOLINE to Cytoscape 3.0.

In particular, I tried to install GASOLINE to Cytoscape 3.0.2 on Mac OS X 10.9.4 via "install from file" and from the App store via App manager. However, I got error messages, and the GASOLINE menu did not appear. I also tried the installation on another machine (Mac OS X snow leopard), but I still got the error.

Other Apps such as MCODE or BiNGO could be installed, thus I do not think that it is the problem of my specific environment.

Therefore, I would like to request authors to solve this problem.

Here is error messages:

The task could not be completed because an error

has occurred.

java.lang.Exception: java.lang.UnsupportedClassVersionError: PluginWrapper : Unsupported major.minor version 51.0

org.cytoscape.work.internal.task.JDialogTaskManager$TaskRunnable.run(JDialogTaskManager.java:307)

java.util.concurrent.Executors$RunnableAdapter.call(Executors.java:439)

java.util.concurrent.FutureTask$Sync.innerRun(FutureTask.java:303)

java.util.concurrent.FutureTask.run(FutureTask.java:138)

java.util.concurrent.ThreadPoolExecutor$Worker.runTask(ThreadPoolExecutor.java:895)

java.util.concurrent.ThreadPoolExecutor$Worker.run(ThreadPoolExecutor.java:918)

java.lang.Thread.run(Thread.java:695)

java.lang.UnsupportedClassVersionError: PluginWrapper : Unsupported major.minor version 51.0

java.lang.ClassLoader.defineClass1(Native Method)

java.lang.ClassLoader.defineClassCond(ClassLoader.java:637)

java.lang.ClassLoader.defineClass(ClassLoader.java:621)

java.security.SecureClassLoader.defineClass(SecureClassLoader.java:141)

java.net.URLClassLoader.defineClass(URLClassLoader.java:283)

java.net.URLClassLoader.access$000(URLClassLoader.java:58)

java.net.URLClassLoader$1.run(URLClassLoader.java:197)

java.security.AccessController.doPrivileged(Native Method)

java.net.URLClassLoader.findClass(URLClassLoader.java:190)

java.lang.ClassLoader.loadClass(ClassLoader.java:306)

java.lang.ClassLoader.loadClass(ClassLoader.java:247)

org.cytoscape.app.internal.manager.SimpleApp.createAppInstance(SimpleApp.java:94)

org.cytoscape.app.internal.manager.SimpleApp.install(SimpleApp.java:173)

org.cytoscape.app.internal.manager.AppManager$1.onFileCreate(AppManager.java:339)

org.apache.commons.io.monitor.FileAlterationObserver.doCreate(FileAlterationObserver.java:379)

org.apache.commons.io.monitor.FileAlterationObserver.checkAndNotify(FileAlterationObserver.java:345)

org.apache.commons.io.monitor.FileAlterationObserver.checkAndNotify(FileAlterationObserver.java:304)

org.cytoscape.app.internal.manager.AppManager.checkForFileChanges(AppManager.java:563)

org.cytoscape.app.internal.manager.AppManager.installApp(AppManager.java:631)

org.cytoscape.app.internal.task.InstallAppFromFileTask.run(InstallAppFromFileTask.java:36)

org.cytoscape.work.internal.task.JDialogTaskManager$TaskRunnable.run(JDialogTaskManager.java:279)

java.util.concurrent.Executors$RunnableAdapter.call(Executors.java:439)

java.util.concurrent.FutureTask$Sync.innerRun(FutureTask.java:303)

java.util.concurrent.FutureTask.run(FutureTask.java:138)

java.util.concurrent.ThreadPoolExecutor$Worker.runTask(ThreadPoolExecutor.java:895)

java.util.concurrent.ThreadPoolExecutor$Worker.run(ThreadPoolExecutor.java:918)

java.lang.Thread.run(Thread.java:695)

I have read this submission. I believe that I have an appropriate level of expertise to confirm that it is of an acceptable scientific standard, however I have significant reservations, as outlined above.

# Response

**Published:** 2014-07-15

**Authors:** Giovanni Micale

The error you encountered was due to the fact that our app was compiled with Java 7, while the last version of Java released by Apple was version 6. So, we recompiled GASOLINE with Java 6 and we tested this new version on all operative systems and it ran without problems. This new version of GASOLINE app (version 1.2) is now available at http://apps.cytoscape.org/apps/gasoline

# Peer review: Referee response for version 1

**DOI:** 10.5256/f1000research.4854.r5404

**Published:** 2014-07-10

**Authors:** Antonio J Perez Pulido, Pablo Mier

**Affiliations:** 1 UPO Genetics Bioinformatics Group, Centro Andaluz de Biología del Desarrollo (CABD), Sevilla, Spain; 2 Universidad Pablo de Olavide, Sevilla, Spain

The article describes a useful plugin for Cytoscape which can be used to align protein interaction networks, including GO annotations. It is an extension of a previous paper where they presented the algorithm. This fact should be clearly mentioned, and the functionality and use of this kind of analysis should also be showed. The authors should also solve the following points: 

**Abstract**

- The abstract should better explain what kind of alignments it makes. It could be confused with sequence local alignments.  

**Introduction**

- What does “GASOLINE” mean? It appears on the web site but not in the actual manuscript.
- “ *Comparing PPI networks of evolutionary distant species can help to understand some mechanisms*”. Indicate what mechanisms.
- “ *Local network alignment aims to compare networks of different species, in order to find conserved protein complexes or pathways*”. Include reference.
- The introduction section is too short. Cytoscape should be introduced. What is it, what are the advantages from it and why was it chosen. They should also explain what a network alignment algorithm is. It is a not a usual algorithm. Its utility must be adequately motivated prior to show the new algorithm. In addition, the introduction should show that GASOLINE algorithm was previously published in another article. Is there any difference with the original algorithm? This is a very important issue because the difference with the previous article must be clear. 

**Implementation**

- “ *the number of iterations of Gibbs sampling in the bootstrap phase / in each extension step of the iterative phase*”. Explain what that is.
- “ *GASOLINE is very fast; it computes the results on 25 networks in a few minutes*”. If it is a result from the previous article, please indicate this or move this to the results.
- What is the ISC score? “ *Index of Structural Conservation*”. But it is not shown in this manuscript. 

**Results**

- The test took 350 seconds on our computers. Perhaps the authors should indicate what kind of computer was used.
- Figure 2 should be used to highlight and describe the different panels and information in them. 

**Conclusions**

- It should indicate potential and practical uses of GASOLINE to solve biological problems. 

**Typos**

- Please, review the syntax. For example, “ *estabilshed*” and some English sentences.

We have read this submission. We believe that we have an appropriate level of expertise to confirm that it is of an acceptable scientific standard, however we have significant reservations, as outlined above.

# Response

**Published:** 2014-09-23

**Authors:** Giovanni Micale

We thank the reviewer for his comments and suggestions.

***The article describes a useful plugin for Cytoscape which can be used to align protein interaction networks, including GO annotations. It is an extension of a previous paper where they presented the algorithm. This fact should be clearly mentioned, and the functionality and use of this kind of analysis should also be showed.***

We clearly state that GASOLINE app is an extension of a previous paper and we provided some examples of application of our tool for annotation transfer and protein prediction.

In what follows we answer to each question.

- ABSTRACT

***The abstract should better explain what kind of alignments it makes. It could be confused with sequence local alignments.***

We clarified that GASOLINE has been implemented for solving local network alignment of PPI networks

- INTRODUCTION

***What does “GASOLINE” mean? It appears on the web site but not in the actual manuscript.***

 We defined the acronym for GASOLINE.

***“Comparing PPI networks of evolutionary distant species can help to understand some mechanisms”. Indicate what mechanisms.***

 We defined what kind of mechanisms (regulation of specific processes of functions within the cell) can be better explained through PPI network alignment.

***“Local network alignment aims to compare networks of different species, in order to find conserved protein complexes or pathways”. Include reference.***

We provided references for the most important local alignment methods.

***The introduction section is too short. Cytoscape should be introduced. What is it, what are the advantages from it and why was it chosen.***

We added a small paragraph, where we briefly introduced Cytoscape, explaining its advantages.

***They should also explain what a network alignment algorithm is. It is a not a usual algorithm. Its utility must be adequately motivated prior to show the new algorithm.***

The network alignment problem has been described more clearly and the differences between local and global alignment methods have been remarked.

***In addition, the introduction should show that GASOLINE algorithm was previously published in another article. Is there any difference with the original algorithm? This is a very important issue because the difference with the previous article must be clear.***

In the "Implementation" section we clarified that there is no technical difference between GASOLINE Cytoscape app and the original algorithm (for which we provided the reference).

- IMPLEMENTATION

***“the number of iterations of Gibbs sampling in the bootstrap phase / in each extension step of the iterative phase”. Explain what that is.***

All the GASOLINE parameters have been better described in the "Introduction" section, where we provided more details of GASOLINE. We indicated that GASOLINE is based on an iterative Gibbs sampling algorithm, which is applied both in the bootstrap and in the iterative phase (extension steps).

***“GASOLINE is very fast; it computes the results on 25 networks in a few minutes”. If it is a result from the previous article, please indicate this or move this to the results.***

This result is taken from the original paper of GASOLINE. However, we decided to remove it from this section and from the whole paper. We specified that GASOLINE is scalable with the number of networks in the “Introduction” section, and we only indicated the running time of GASOLINE for the test reported in the “Results” section.

***What is the ISC score? “Index of Structural Conservation”. But it is not shown in this manuscript.***

We defined what the ISC score is and how it is used to evaluate the quality of local alignments.

- RESULTS

***The test took 350 seconds on our computers. Perhaps the authors should indicate what kind of computer was used.***

We indicated the technical characteristics of the machine used for the tests.

***Figure 1 should be used to highlight and describe the different panels and information in them.***

We revised Figure 1 highlighting and describing each GASOLINE panel.

- CONCLUSIONS

***It should indicate potential and practical uses of GASOLINE to solve biological problems.***

We give few more details concerning the potential applications of GASOLINE in annotation transfer and protein function prediction.

- TYPOS

***Please, review the syntax. For example, “estabilshed” and some English sentences.***

Fixed.
